# Young female participants show blunted placebo effects associated with blunted responses to a cue predicting a safe stimulus in the right dorsolateral prefrontal cortex

**DOI:** 10.3389/fnins.2022.1001177

**Published:** 2022-10-03

**Authors:** Yudai Iwama, Kouichi Takamoto, Daisuke Hibi, Hiroshi Nishimaru, Jumpei Matsumoto, Tsuyoshi Setogawa, Hisao Nishijo

**Affiliations:** ^1^System Emotional Science, Faculty of Medicine, University of Toyama, Toyama, Japan; ^2^Department of Sport and Health Sciences, Faculty of Human Sciences, University of East Asia, Shimonoseki, Japan; ^3^Department of Anesthesiology, Faculty of Medicine, University of Toyama, Toyama, Japan; ^4^Research Center for Idling Brain Science (RCIBS), University of Toyama, Toyama, Japan

**Keywords:** dorsolateral prefrontal cortex, hemodynamic responses, placebo analgesia, sex, aging

## Abstract

Discrimination of cues predicting non-nociceptive/nociceptive stimuli is essential for predicting whether a non-painful or painful stimulus will be administered and for eliciting placebo/nocebo (pain reduction/pain enhancement) effects. Dysfunction of the neural system involved in placebo effects has been implicated in the pathology of chronic pain, while female sex is one of the important risk factors for development of chronic pain in young adults. The dorsolateral prefrontal cortex (dl-PFC) is suggested to be involved in placebo effects and is sensitive to sex and age. In this study, to examine the neural mechanisms by which sex and age alter placebo and nocebo effects, we analyzed cerebral hemodynamic activities in the dl-PFC in different sex and age groups during a differential conditioning task. During the training session, two different sounds were followed by low- and high-intensity electrical shocks. In the following recording session, electrical shocks, the intensity of which was mismatched to the sounds, were occasionally administered to elicit placebo and nocebo effects. In young female participants, both placebo effects and hemodynamic responses to the conditioned sounds in the right dl-PFC were significantly lower than those in elderly female participants, while there were no age differences in male participants. The hemodynamic responses to the sound paired with the safe stimulus in the right dl-PFC were significantly correlated with placebo effects, except in the young female group. These results suggest that blunted placebo effects in the young female participants are ascribed to blunted responses to the sound associated with the safe stimulus in the right dl-PFC, and that sex- and age-related factors may alter the responsiveness of the right dl-PFC to associative cues predicting a safe stimulus.

## Introduction

Pain experience is influenced by various psychological factors such as learning and prediction. For example, predicting weak pain reduces subjective pain (placebo effects), whereas predicting severe pain increases subjective pain (nocebo effects). These alterations in pain sensation are suggested to be mediated by the descending pain modulation system that regulates nociceptive information processing in the spinal cord (Colloca and Grillon, 2014). The dorsolateral prefrontal cortex (dl-PFC) is involved in memory, prediction, and pain cognition. The dl-PFC, as an upstream brain region to trigger the descending pain modulation system, plays an important role in prediction-based pain modulation (Wager, 2005; Atlas and Wager, 2012). Placebo effects are associated with the prediction of analgesic effects and can be experimentally induced by the presentation of specific cues associated with non-noxious weak pain (Wager and Atlas, 2015). The dl-PFC activity has been reported to increase during presentation of cues predicting weak pain (i.e., safe stimuli), and this activity correlates with placebo effects (Wager et al., 2004; Hibi et al., 2020). Normal functioning of the dl-PFC is important in pain free health and pain management since insufficient activation of the descending pain modulatory system may induce the onset of chronic pain (Ossipov et al., 2014; Zortea et al., 2019). Consistently, patients with chronic neuropathic pain due to postherpetic neuralgia displayed blunted responses to a cue associated with a safe stimulus in the dl-PFC (Hibi et al., 2020).

It has been reported that female sex is one of the important risk factors for development of chronic pain in young adults ranging from 18 to 29 years old (Brown et al., 2021). This suggests that neural mechanisms to predict a safe stimulus might be less evident in young females. Consistently, meta-analysis studies have suggested that placebo and nocebo effects are affected by sex and age, although inconsistent results were reported (Weimer et al., 2015; Vambheim and Flaten, 2017; Enck and Klosterhalfen, 2019). The previous studies cited in these meta-analysis studies investigated effects of sex and age separately, which might lead to inconsistent results if sex effects are dependent on age. Furthermore, neural mechanisms underlying sex-specific age-related effects on placebo analgesia are unknown. Previous studies have suggested that the dl-PFC is functionally and morphologically altered by sex-specific and age-dependent processes (Jacobs and Goldstein, 2018). Thus, cognitive functions, such as working memory associated with the dl-PFC and activity in the dl-PFC during cognitive tasks, are affected by sex and age (Jacobs et al., 2017; Jacobs and Goldstein, 2018). These findings suggest that sex-specific and age-dependent alterations in the dl-PFC may affect prediction-based pain modulation. In this study, we hypothesized that the predictive activity in the dl-PFC is altered in a sex-specific and age-dependent manner and is associated with sex-specific and age-dependent alteration of placebo effects. To test this hypothesis, we examined cerebral activity in the dl-PFC with near-infrared spectroscopy (NIRS) during pain prediction in different sex and age groups using a differential conditioning task in which two types of sounds were paired with low- and high-intensity electric shocks. Here, we report sex-specific age-dependent changes in the hemodynamic responses to sound paired with low-intensity shock in the right dl-PFC, which were linked to sex-specific age-dependent alterations of placebo effects.

## Materials and methods

### Participants

A total of 60 healthy participants were used in the current study and divided by sex and age into four groups: elderly male group [*n* = 15; mean age ± standard error of mean (SEM), 66.2 ± 2.1 years], elderly female group (*n* = 15; 66.3 ± 1.7 years), young male group (*n* = 15; 22.3 ± 0.5 years), and young female group (*n* = 15; 22.5 ± 0.6 years). Cognitive function in the elderly groups was assessed using the Japanese version of the Mini-Mental State Examination (J-MMSE) (Sugishita et al., 2016) to confirm that they did not have dementia. The experimental protocols in this study were consistent with the guidelines set by the Declaration of Helsinki and were reviewed and approved by the Ethics Assessment Committee for Research with Humans at the University of Toyama (permit no.: R2017136). All participants provided written informed consent.

### Experimental procedures

In this study, we used a differential conditioning task according to our previous study (Hibi et al., 2020). The current intensity of the electrical shock was determined for each participant. Conditioning training using the differential conditioning task was then conducted for participants to learn the relationships between the two different sounds and two painful stimuli with different current intensities. Finally, the cerebral hemodynamic activity in the right and left dl-PFC was measured during the differential conditioning task using NIRS (NIRS recording session). In 75% of trials in the NIRS recording session, the same pairs of sounds and painful stimuli as in the conditioning training were presented. In the remaining 25% of trials, painful stimuli mismatched to the sounds were presented (mismatched condition) to elicit placebo and nocebo effects.

#### Determination of current intensity of electrical stimulation

In this study, 5-Hz sine-wave electrical stimulation was used as a painful stimulus generated by an electrical stimulator (Neurometer, Neurotron Inc., Baltimore) and delivered through electrodes set on the dorsal surface of participants’ left hand. While the current intensity of electrical stimulation was gradually increased, participants were required to respond by saying “yes” when the pain on the dorsal hand reached the maximum that they could tolerate (maximum current intensity for highest endurable pain). For the differential conditioning task, two current intensities of electrical shock were set according to the maximum current intensity in each participant: 50% of the maximum current intensity as low-intensity stimulation (LS) and 90% as high-intensity stimulation (HS). After determination of the maximum current intensity, participants received LS and HS to confirm that they felt low- and high-intensity pain, respectively, when the stimuli were delivered.

#### Conditioning training with the differential conditioning task

The 20 trials were presented as the conditioning training for participants to learn the relationships between the two types of sounds and the corresponding intensity of the electrical stimulation. Low-frequency (LF, 500 Hz) sound at 60 dB and high-frequency (HF, 2000 Hz) sound at 87 dB were presented as predictive cues, followed by LS and HS, respectively. Each trial was composed of a sound presentation (5 s), delay period (5 s), electrical shock (5 s), and rest (10 s). The 10 trials for each pair of LF sound-LS and HF sound-HS were presented randomly. After electrical stimulation in each trial, participants reported their subjective pain scores according to a visual analog scale (VAS) ranging from 0 (no pain) to 100 (highest endurable pain).

#### NIRS recording session

In this session, cerebral hemodynamic activity was monitored during the differential conditioning task. Each trial was composed of a sound presentation (5 s), delay period (5 s), electrical shock (5 s), and rest (variable between 20 and 30 s). To prevent anticipatory brain activity for sound onset, the rest period was randomized (mean rest period, 24.84 ± 0.71 s). According to the previous study (Hibi et al., 2020), each participant received 32 trials consisting of the following four conditions: LF-LS (matched) condition (LF sound paired with LS), 13 trials; HF-HS (matched) condition (HF sound paired with HS), 11 trials; LF-HS (mismatched) condition (LF sound paired with HS), four trials; and HF-LS (mismatched) condition (HF sound paired with LS), four trials. The four conditions were presented pseudo-randomly. After completion of each electrical stimulation, participants rated their subjective pain scores using VAS.

**FIGURE 1:**
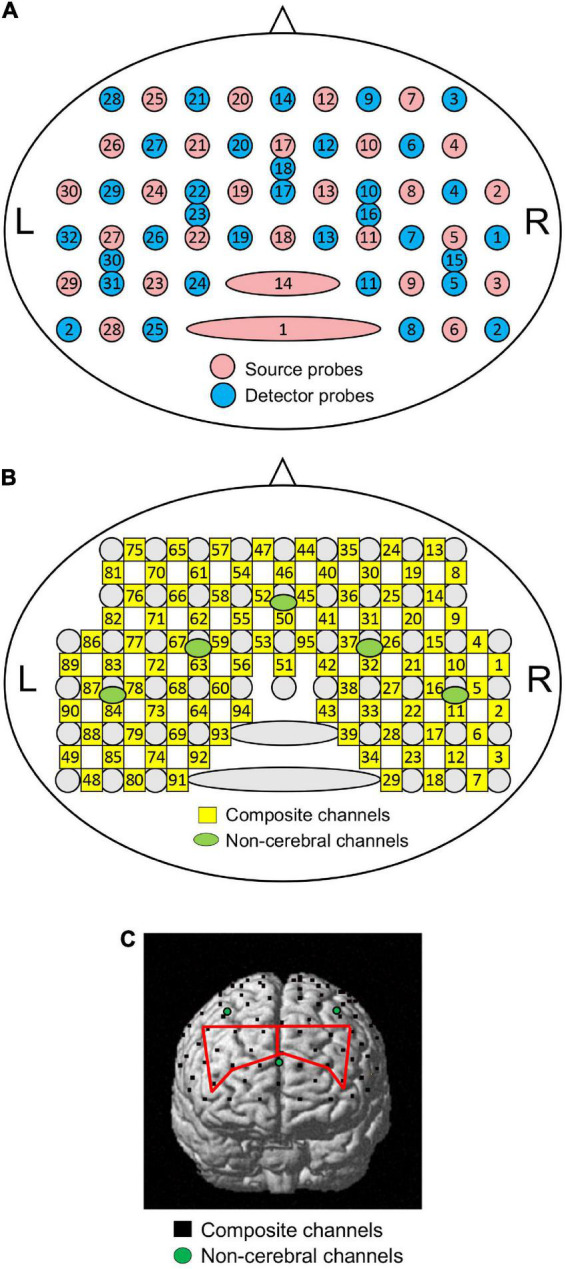
Schematic presentation of NIRS probe and channel locations. **(A)** Arrangement of source and detector probes in a NIRS head cap. Numbers in red and blue circles indicate source and detector numbers, respectively. L, left; R, right. **(B)** Arrangement of NIRS channels. Numbers in green circles indicate composite channel numbers; numbers in green circles indicate non-cerebral channel numbers. **(C)** Identification of NIRS channel locations in one participant. Black dots indicate composite channels; green circles indicate non-cerebral channels. Regions of interest (ROIs) [the right and left dorsolateral prefrontal cortices (dl-PFC) are indicated by red lines.

Two NIRS instruments (OMM 3000, Shimadzu Inc., Kyoto) were used in this study. A head cap with receptacles for NIRS probes was set on the head of participants, and its position was adjusted such that the lowest and most anterior NIRS probes were located on the FP2-FP10 line in the electroencephalogram 10–20 system (Jurcak et al., 2007). A total of 28 optical sources and 32 detector probes were attached to the head cap (Figure 1; Hibi et al., 2020; Kobayashi et al., 2021). Twenty-seven and five detector probes were placed at 3.0 and 1.5 cm from the source probes, respectively (Figure 1A). The measurement locations (channels) were assumed to be located at the midpoints between the detector and source probes (Figure 1B).

Changes in oxygenated hemoglobin (HbO), deoxygenated Hb (HbR), and total Hb concentrations were measured in each channel to assess brain hemodynamics. Hemodynamic signals from the detector-source pairs with an inter-probe distance greater than 3 cm reflect hemodynamic changes in both the brain (cortex) and structures outside the brain (scalp, skull, and cerebrospinal fluid) (composite channels). Hemodynamic signals from the detector-source pairs with inter-probe distances less than 1.5 cm mainly reflect hemodynamic changes in the structures outside the brain (non-cerebral channels). To remove hemodynamic signals from structures outside the brain, a probe arrangement with multiple inter-probe distances was used, as previously described (Saager et al., 2011; Ishikuro et al., 2014; Sato et al., 2016; Nakamichi et al., 2018; Hibi et al., 2020; Kobayashi et al., 2021). Signals from 95 composite and five non-cerebral channels were recorded (Figure 1B). After the measurements, three-dimensional (3D) coordinates of all the probes were measured using FASTRAK^®^ (Polhemus Inc., Colchester, Vermont).

### Data analysis

#### Baseline characteristics and subjective pain

Comparison of the baseline characteristics between the groups (age and MMSE) was performed using the Mann–Whitney *U* test, while the current intensity of highest endurable electrical stimulation was compared among the four groups using one-way ANOVA.

Mean VAS pain scores of HS were statistically compared with repeated-measures three-way ANOVA with age (young vs. elderly), sex (male vs. female), and condition [matched (HS following the HF sound: HF-HS condition) vs. mismatched (HS following the LF sound: LF-HS condition)] as factors. Mean VAS pain scores of LS were similarly compared with repeated-measures three-way ANOVA with age (young vs. elderly), sex (male vs. female), and condition [matched (LS following the LF sound: LF-LS condition) vs. mismatched (LS following the HF sound: HF-LS condition)] as factors. Pain reduction index explaining placebo effects were defined as differences in VAS pain scores of HS between the matched and mismatched conditions (VAS pain score in the HF-HS condition – VAS pain score in the LF-HS condition): the larger pain reduction index is associated with the larger placebo effects. Pain enhancement index explaining nocebo effects were defined as differences in VAS pain scores of LS between the mismatched and matched conditions (VAS pain score in the HF-LS condition – VAS pain score in the LF-LS condition): the larger pain enhancement index is associated with the larger nocebo effects. Pain reduction and enhancement indices were statistically compared with two-way ANOVA with age (young vs. elderly) and sex (male vs. female) as factors.

#### NIRS data analysis

NIRS data were bandpass filtered at 0.01–0.1 Hz for removal of physiological noises (cardiac activity, respiration, etc.) and baseline drift correction (Tong et al., 2011; Yasumura et al., 2014). In addition, the systemic component of hemodynamic changes was removed from the resultant NIRS signals. Hemodynamic signals from the non-cerebral channels are thought to contain predominantly systemic hemodynamic changes in non-cerebral structures. To remove systemic hemodynamic changes from the signals in the composite channels, signals from the five non-cerebral channels were analyzed using a spatial filtering algorithm with principal component analysis (PCA) (Zhang et al., 2016, 2017). The first two principal components of the hemodynamic signals in the five non-cerebral channels were removed from the signals in the composite channels.

In this study, the resultant HbO data were further analyzed for cerebral hemodynamic activity since signal-to-noise ratios are higher in HbO than in HbR (Tian et al., 2012; Ding et al., 2014; Sato et al., 2016), and HbR signals are affected by changes in venous blood volumes and venous blood oxygenation (Hoshi et al., 2001). Hemodynamic HbO responses to LF and HF sounds were summed and averaged. The averaged responses were corrected for mean baseline HbO levels during 5 s prior to the sound onset. Finally, HbO responses to the sounds were estimated as effect sizes: effect size = [(mean HbO levels during 10 s after sound onset) – (mean baseline HbO levels during 5 s prior to sound onset)]/[standard deviation (SD) of HbO levels during 5 s prior to sound onset].

To estimate the locations of the composite channels in the brain, the channel coordinates were calculated from the 3D coordinates of the NIRS probes in each participant and converted to Montreal Neurological Institute (MNI) coordinates (Tsuzuki et al., 2007). Subsequently, anatomical positions and Brodmann areas (BA) of the composite channels in each participant were identified using MRIcro software (Figure 1C).^1^ In this study, two brain regions [left and right dl-PFC (BA 9, 46)] were focused on as regions of interest (ROIs). In each participant, the mean effect sizes of cerebral HbO responses to each sound were estimated for each ROI. Finally, mean cerebral HbO responses (effect sizes) in each ROI to each sound were compared with repeated-measures three-way ANOVA with age (young vs. elderly), sex (male vs. female), and condition (LF vs. HF sounds) as factors.

#### Correlation analyses

The relationships between pain reduction and enhancement indices and predictive variables [sex, age, and hemodynamic (HbO) responses (effect sizes) to LF and HF sounds in the right and left dl-PFC] were analyzed using Pearson’s correlation and multiple regression analysis with the forced entry as well as stepwise methods.

### Statistical analyses

Data are presented as mean ± SEM. Assumption of normal distribution of the results was tested using the Kolmogorov–Smirnov test. Homogeneity of variance for one-way ANOVA was checked using Brown–Forsythe’s modification of the Levene’s test. In repeated-measures three-way ANOVA, homogeneity of variance was assessed with Mauchly’s test of sphericity. Statistical significance was set at *p* < 0.05. All statistical tests were performed with SPSS Statistics ver. 28 (SPSS Inc., Chicago).

## Results

### Baseline characteristics

Baseline characteristics (age, current intensity at highest endurable pain, and MMSE score) of the four groups are shown in Table 1. Comparison between the elderly male and female groups indicated that there were no significant differences in age [Mann–Whitney *U* test: *p* = 0.806, effect size (*r*) = –0.028] and MMSE scores [Mann–Whitney *U* test: *p* = 0.098, effect size (*r*) = –0.208]. Furthermore, the mean age did not differ significantly between the young male and female groups [Mann–Whitney *U* test: *p* = 0.838, effect size (*r*) = –0.024]. However, the mean current intensity for highest endurable pain differed significantly among the four groups [one-way ANOVA: *F*(3, 56) = 5.865, *p* = 0.0014]. Subsequent comparisons indicated that current intensity was significantly greater in the young male group than that in the elderly male (Bonferroni test, *p* = 0.0173) and elderly female (Bonferroni test, *p* = 0.0017) groups.

**TABLE 1:** Baseline data of the four participant groups.

	Elderly male (*n* = 15)	Elderly female (*n* = 15)	Young male (*n* = 15)	Young female (*n* = 15)
Age (years)	66.2 ± 2.1	66.3 ± 1.7	22.3 ± 0.5	22.5 ± 0.6
Current intensity for highest endurable pain (mA)	2.12 ± 0.32	1.68 ± 0.30	3.93 ± 0.41^#^	3.01 ± 0.55
MMSE	29 ± 0.29	29.5 ± 0.21	–	–

Values are means ± standard error (SE); MMSE, mini-mental state examination. ^#^, Significant difference from the elderly male (*p* < 0.05) and elderly female (*p* < 0.01) groups.

### Comparison of subjective pain scores

**FIGURE 2:**
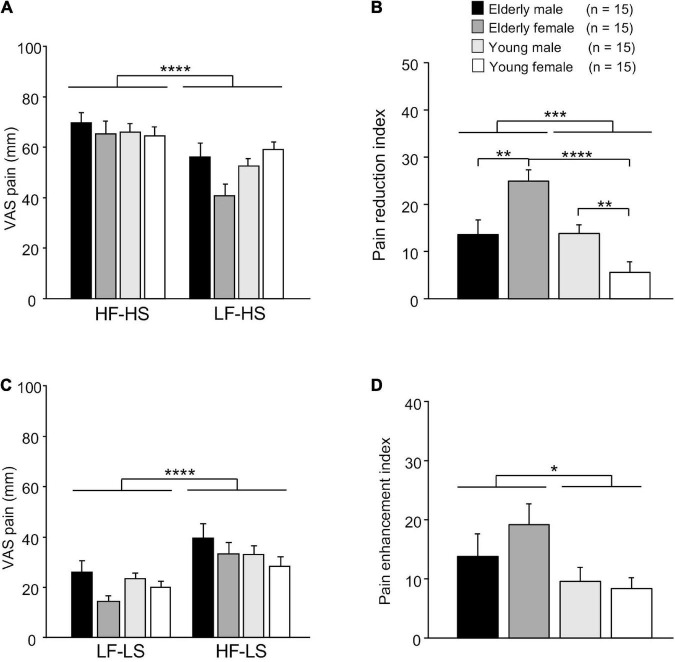
Comparison of VAS scores of electrical shocks among groups. **(A)** Comparison of VAS scores of high-intensity electrical shock (HS) following the two different sounds. VAS, visual analog scale; HF-HS, HS following the high frequency (HF) sound; LF-HS, HS following the low frequency (LF) sound. **(B)** Comparison of pain reduction indices (difference in VAS pain scores of HS = VAS in HF-HS – VAS in LF-HS). **(C)** Comparison of VAS scores of low-intensity electrical shock (LS) following the two different sounds. LF-LS, LS following the LF sound; HF-LS, LS following the HF sound. **(D)** Comparison of pain enhancement indices (difference in VAS pain scores of LS = VAS in HF-LS – VAS in LF-LS). Error bars indicate the SEM. *, **, ***, ****, *p* < 0.05, 0.01, 0.001, 0.0001, respectively.

The mean subjective pain scores of LS and HS in each condition (matched vs. mismatched) are shown in Figure 2. Figure 2A shows mean VAS pain scores of HS in the matched (HF-HS) and mismatched (LF-HS) conditions, and analyzed with a repeated-measures three-way ANOVA with age (elderly vs. young), sex (male vs. female), and condition (matched vs. mismatched) as factors. The results showed a significant main effect of the condition [*F*(1, 112) = 24.405, *p* < 0.0001] and a significant interaction between age and sex [*F*(1, 112) = 4.465, *p* = 0.037], but no significant main effects of age [*F*(1, 112) = 0.779, *p* = 0.379] and sex [*F*(1, 112) = 1.413, *p* = 0.237]. These findings indicate that VAS pain scores were smaller in the LF-HS mismatched condition than in the HF-HS matched condition across the four groups, further indicating that placebo effects were evident across the four groups.

Pain reduction indices (differences in VAS pain scores = VAS score in the HF-HS matched condition – VAS score in the LF-HS mismatched condition) in the four groups are shown in Figure 2B. A statistical comparison by two-way ANOVA revealed that there were significant main effect of age [*F*(1, 28) = 15.728, *p* = 0.0003], and significant interaction between age and sex *F*(1, 28) = 16.644, *p* = 0.0004]. However, there was no significant main effect of sex [*F*(1, 28) = 0.432, *p* = 0.516]. Subsequent comparisons indicated that pain reduction indices were significantly greater in the elderly female group than in the elderly male group (Bonferroni test, *p* = 0.007) and young female group (Bonferroni test, *p* < 0.0001). Furthermore, pain reduction indices were significantly greater in young male group than in the young female group (Bonferroni test, *p* = 0.006).

Figure 2C shows mean VAS pain scores of LS in the matched (LF-LS) and mismatched (HF-HS) conditions, and analyzed with a repeated-measures three-way ANOVA with age, sex, and condition as factors (Figure 2C). The results showed significant main effects of condition [*F*(1, 112) = 20.580, *p* < 0.0001] and sex [*F*(1, 112) = 4.832, *p* = 0.030]. However, there were no significant main effect of age [*F*(1, 112) = 0.549, *p* = 0.460], nor significant interactions in all possible combinations of the factors (data not shown). These findings indicate that VAS pain scores were greater in the HF-LS mismatched condition than in the LF-LS matched condition across the four groups, further indicating that there was a significant nocebo effect across all participants.

Pain enhancement indices (differences in VAS pain scores = VAS score in the HF-LS mismatched condition – VAS score in the LF-LS matched condition) in the four groups are shown in Figure 2D. A statistical comparison by two-way ANOVA revealed a significant main effect of age [*F*(1, 28) = 4.951, *p* = 0.034]. However, there was no significant main effect of sex [*F*(1, 28) = 0.639, *p* = 0.431], nor significant interaction between age and sex [*F*(1, 28) = 0.931, *p* = 0.343].

### Cerebral hemodynamic responses in the dorsolateral prefrontal cortex

**FIGURE 3:**
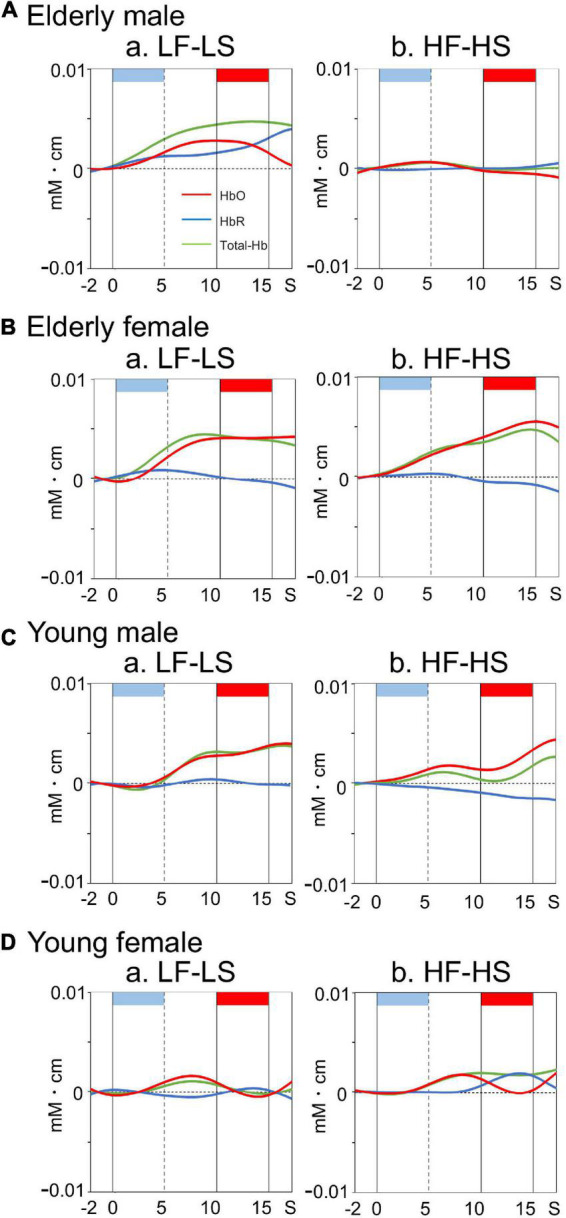
Examples of time courses of cerebral hemodynamic activity in the right dl-PFC during the differential conditioning task in elderly male **(A)**, elderly female **(B)**, young male **(C)**, and young female **(D)** groups. Hemodynamic signals are recorded from Ch 41 [MNI coordinates: (22, 57, 37) mm] in an elderly male **(A)**, Ch 41 [MNI coordinates: (30, 55, 34) mm] in an elderly female **(B)**, Ch 41 [MNI coordinates: (29, 55, 34) mm] in a young male **(C)**, and Ch 41 [MNI coordinates: (27, 60, 27)] in a young female **(D)** participant. Light blue and red rectangles at the top of each graph indicate sound presentation and electrical shock periods, respectively. Red, blue, and green lines indicate HbO, HbR, and Total-Hb, respectively. Other descriptions are as for Figure 2.

**FIGURE 4:**
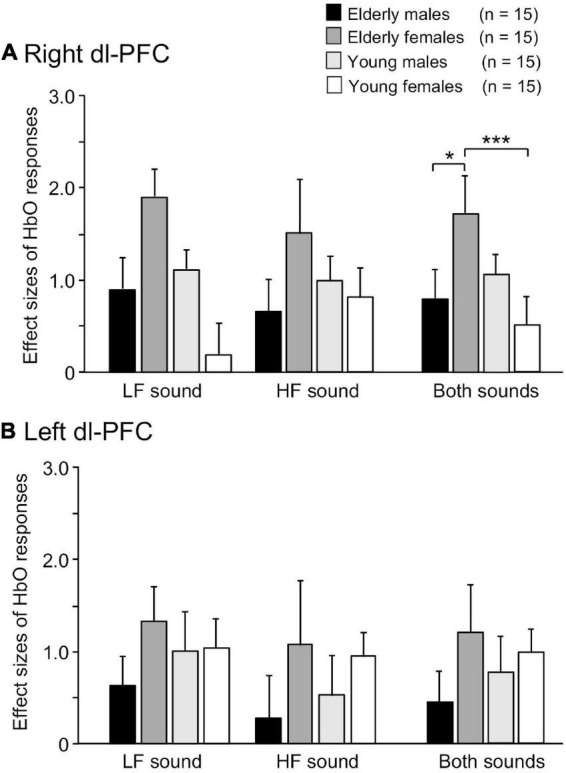
Comparison of effect sizes of cerebral HbO responses in the right **(A)** and left **(B)** dl-PFC. *, ***, *p* < 0.05, 0.001, respectively.

Examples of time courses of changes in HbO, HbR, and total-Hb concentrations in the right dl-PFC during the task are shown in Figure 3. Compared to participants in the elderly male, elderly female, and young male groups (Figures 3A–C), a participant in the young female group showed blunted HbO responses during and after sound presentation in the LF-LS condition (Figure 3D). Mean effect sizes of the cerebral HbO responses to LF and HF sounds in the right dl-PFC are shown in Figure 4A, and analyzed with a repeated-measures three-way ANOVA with age (young vs. elderly), sex (male vs. female), and condition (LF vs. HF sounds) as factors. The results indicated a significant interaction between age and sex [*F*(1, 112) = 8.719, *p* = 0.004], but no significant main effects of age [*F*(1, 112) = 3.433, *p* = 0.067], sex [*F*(1, 112) = 0.572, *p* = 0.451], and condition [*F*(1, 112) = 0.011, *p* = 0.916]. The remaining interactions in all possible combinations of the factors were not statistically significant (data not shown). *Post hoc* comparisons revealed that mean effect sizes of the cerebral HbO responses to both sounds in the right dl-PFC were significantly greater in the elderly female group than in the elderly male group (Bonferroni test, *p* = 0.010) and in the young female group (Bonferroni test, *p* = 0.0009).

Conversely, a statistical analysis of the effect sizes of the cerebral HbO responses to the sounds in the left dl-PFC by repeated-measures three-way ANOVA (age × sex × condition) indicated that there were no significant main effects of age [*F*(1, 112) = 0.028, *p* = 0.867], sex [*F*(1, 112) = 2.439, *p* = 0.121], and condition [*F*(1, 112) = 0.881, *p* = 0.350] and no significant interactions in all possible combinations of the factors (data not shown) (Figure 4B).

### Relationships between psychological data and hemodynamic responses

**FIGURE 5:**
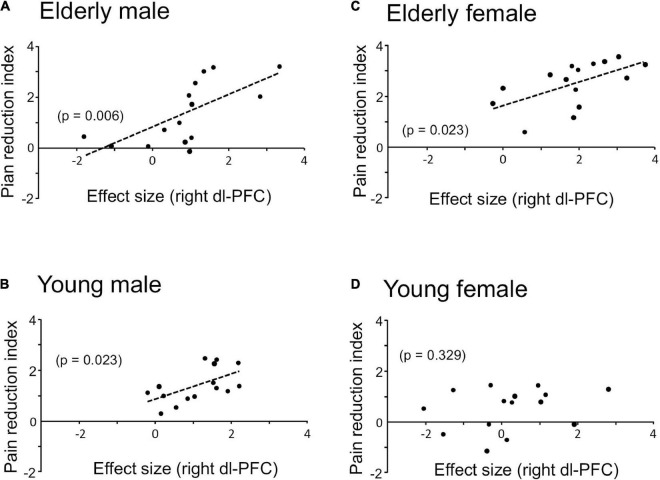
Relationships between pain reduction indices and effect sizes of cerebral HbO responses to the LF sound in the right dl-PFC in elderly male **(A)**, elderly female **(B)**, young male **(C)**, and young female **(D)** groups. Significant positive correlations are observed in all groups except the young female group.

**FIGURE 6:**
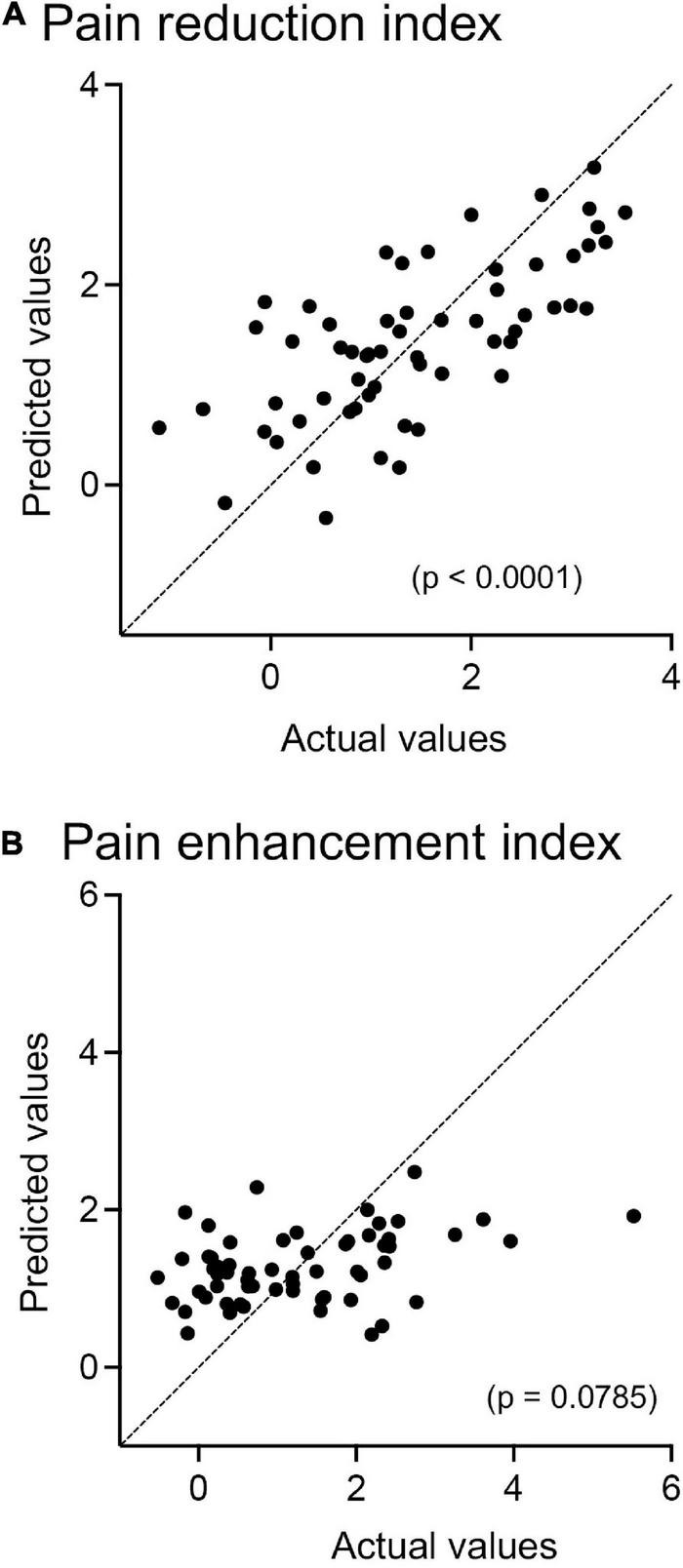
Relationships between predicted and actual pain-reduction **(A)** and pain-enhancement **(B)** indices in multiple regression analyses with the forced entry method. **(A)** The model with the four predictor variables (sex, age, effect sizes of cerebral HbO responses to the LF sound in the right dl-PFC, and effect sizes of cerebral HbO responses to the LF sound in the left dl-PFC) significantly predicts pain reduction indices (*p* < 0.0001). **(B)** The model with the four predictor variables (sex, age, effect sizes of cerebral HbO responses to the HF sound in the right dl-PFC, and effect sizes of cerebral HbO responses to the HF sound in the left dl-PFC) does not significantly predict pain enhancement indices (*p* = 0.0785).

Since the prediction of weak (safe) pain plays an essential role in placebo effects (see section “Introduction”), dl-PFC responses to LF sounds associated with LS are thought to be involved in placebo effects. The relationships between the pain reduction indices (differences in VAS pain scores = HF-HS matched condition – LF-HS mismatched condition) and effect sizes of the cerebral HbO responses to the LF sound in the right dl-PFC are shown in Figure 5. A statistical analysis of the data in each group with Pearson’s correlation revealed significant positive correlations between pain reduction indices and effect sizes of the cerebral HbO responses in the right dl-PFC in the elderly male (*r* = 0.669, *p* = 0.006, not corrected) (A), elderly female (*r* = 0.582, *p* = 0.023, not corrected) (B), and young male (*r* = 0.580, *p* = 0.023, not corrected) (C) groups. When the data were corrected for multiple comparisons with Holm method, the elderly male (*p* = 0.024) and elderly female (*p* = 0.046) groups showed significant correlations, while the young male group showed a marginally significant correlation (*p* = 0.069). These results indicated that greater pain reduction indices (i.e., placebo effects) was attributed to greater cerebral HbO responses in the right dl-PFC. However, in the young female group, pain reduction indices were not significantly related to effect sizes of cerebral HbO responses to the LF sound in the right dl-PFC (*r* = 0.271, *p* = 0.329, not corrected) (D). In contrast, in the left dl-PFC, there were no significant correlations between pain reduction indices and effect sizes of cerebral HbO responses to the LF sound in all participant groups (Supplementary Figure 1). Data of the four groups were further analyzed by a multiple regression analysis with the forced entry method to predict pain reduction indices from the four predictor variables (sex, age, effect sizes of cerebral HbO responses to the LF sound in the right dl-PFC, and effect sizes of cerebral HbO responses to the LF sound in the left dl-PFC). The model with these variables significantly predicted pain reduction indices [*F*(4, 55) = 12.76, *p* < 0.0001, *R*^2^ = 0.481] (Figure 6A). Among the four predictive variables, only effect sizes of the cerebral HbO responses to LF sound in the right dl-PFC significantly contributed to the prediction (β = 0.574, *p* < 0.0001). To identify possible predictors of the pain reduction index, the same data set was analyzed using a stepwise multiple linear regression based on *p*-value. The stepwise analysis with *p* < 0.01 indicated only one predictor (effect sizes of the cerebral HbO responses to LF sound in the right dl-PFC) (β = 0.658, *p* < 0.0001), while the analysis with *p* < 0.05 indicated effect sizes of the cerebral HbO responses to LF sound in the right dl-PFC (β = 0.599, *p* < 0.0001) as well as age (β = 0.207, *p* = 0.044) as predictors. Thus, the stepwise multiple regression analysis also indicated a significant contribution of effect sizes of the cerebral HbO responses to LF sound in the right, but not left, dl-PFC to predict the pain reduction index.

Since the prediction of strong pain plays an essential role in nocebo effects (see section “Introduction”), dl-PFC responses to the HF sound associated with HS are supposed to be involved in nocebo effects if the dl-PFC is involved in nocebo effects. However, statistical analyses of the data with Pearson’s correlation revealed that pain enhancement indices (nocebo effects) were not significantly correlated with effect sizes of cerebral HbO responses to the HF sound in the right dl-PFC in any participant group (Supplementary Figure 2) or with those in the left dl-PFC in all participant groups (Supplementary Figure 3). Data of the four groups were further analyzed by a multiple regression analysis to predict the pain enhancement indices from the four predictor variables (sex, age, effect sizes of cerebral HbO responses to the HF sound in the right dl-PFC, and effect sizes of cerebral HbO responses to the HF sound in the left dl-PFC). However, the model with these variables did not significantly predict pain enhancement indices [*F*(4, 55) = 2.222, *p* = 0.079, *R*^2^ = 0.139] (Figure 6B).

## Discussion

### Neural mechanisms mediating placebo and nocebo effects

The results of this study indicated that pain reduction indices were positively related to effect sizes of cerebral HbO responses to the LF sound paired with the LS in the right dl-PFC, and greater VAS pain reduction was linked to greater hemodynamics in the right dl-PFC. Consistent with the present results, in patients with postherpetic neuralgia, placebo effects were shown to be related to effect sizes of cerebral HbO responses to a cue associated with a safe stimulus (low-intensity shock) in the right dl-PFC, and clinical symptoms of chronic pain were negatively related to effect sizes of cerebral HbO responses to the safe cue in the right dl-PFC (Hibi et al., 2020). The present results extend the understanding of the role of the right dl-PFC in healthy controls.

Consistent with the present results, the right dl-PFC is active during the prediction of analgesia in a task to associate conditioned stimuli with painful stimuli, and its right dl-PFC activity is correlated with analgesic effects (Lui et al., 2010). Non-invasive electrical activation of the right dl-PFC augmented placebo effects (Egorova et al., 2015; Tu et al., 2021), while placebo effects were suppressed by inhibitory stimulation of the right dl-PFC (Krummenacher et al., 2010). These previous findings strongly suggest that activity in the right dl-PFC in response to associative cues predicting low-intensity pain stimuli is involved in placebo effects. As the dl-PFC projects to the descending pain modification system (Ong et al., 2019; Zortea et al., 2019), the right dl-PFC may exert placebo effects through the descending pain modification system. On the other hand, pain reduction indices were not significantly related to effect sizes of cerebral HbO responses to the LF sound in the left dl-PFC. However, this finding does not deny the role of the left dl-PFC in placebo analgesia. A meta-analysis study has suggested that activity of the PFC is heterogeneous depending on placebo-induction methods (Zunhammer et al., 2021), and the left dl-PFC may be involved in placebo analgesia in placebo-induction methods different from the present study.

Visual analog scale pain scores in the HF-LS condition were greater than those in the LF-LS condition across the four groups, indicating that Nocebo effects were significantly estimated in the present study. Furthermore, pain enhancement indices were greater in the elderly groups than in the young groups, consistent with previous studies (see a review by Kravvariti et al., 2021). However, pain enhancement indices (nocebo effects) were not significantly related to effect sizes of cerebral HbO responses to the HF sound in the right and left dl-PFC. Previous studies have suggested that deep brain regions, such as the insular cortex, hippocampus, and anterior cingulate cortex, are involved in nocebo effects (Kong et al., 2008; Rodriguez-Raecke et al., 2010; Blasini et al., 2017). Consistently, a recent study reported that two separate systems encode the prediction of safe and painful situations: cortical regions, including the dl-PFC, are involved in learning the relationships between a cue and absence of pain, while the subcortical and limbic systems are involved in learning the relationships between a cue and pain (Jepma et al., 2022). In this study, cerebral HbO responses to sounds were measured from the cerebral cortex (dl-PFC), and consequently, no significant relationships between cerebral hemodynamic responses and pain enhancement indices (nocebo effects) were observed. Future studies are required to analyze sex and age effects in deep or subcortical brain regions related to nocebo effects using functional magnetic resonance imaging or other methods that can measure activity in deep brain regions.

### Characteristics of young female participants regarding placebo effects

In this study, there was a significant age difference in the female participants in two aspects: the young female group showed smaller pain reduction indices than did the elderly female group, and there was no significant relationship between effect sizes of the cerebral HbO responses to LF sound paired with LS in the right dl-PFC and pain reduction indices in the young female participants, while the elderly female participants showed a significant correlation. In contrast, there were no age differences between the two male participants. These findings provide neuroscientific evidence that female sex is a risk factor for chronic pain in young adults (see section “Introduction”). As discussed in the previous section, the dl-PFC is involved in working memory, and its activation based on associative learning is associated with the prediction of a safe stimulus (Wager et al., 2004; Wager and Atlas, 2015). The present results and those of these previous studies suggest that there may be age-related differences in psychological characteristics between the two female groups, which may affect responses to associative cues (especially LF sound) in the dl-PFC in female participants, leading to differences in placebo effects between the two female groups (see below).

Affective factors, such as anxiety, have been reported to closely interact with pain perception and placebo effects (Ploghaus et al., 2001; Atlas, 2021). Anxiety in female individuals decreases with age (Jacobs et al., 2017), while the proportion of young female individuals with anxiety has increased rapidly in recent years (Calling et al., 2017). Furthermore, the incidence of anxiety disorders is higher in younger female individuals and lower in elderly female individuals (Henderson et al., 1998; Remes et al., 2016). These previous studies suggest that young female individuals may be more prone to anxiety, which is one of the age-related differences in female individuals. On the other hand, discrimination of cues associated with nociceptive and non-nociceptive stimuli is impaired by anxiety (Dibbets et al., 2015). In female participants, the ability to discriminate associative cues is affected by anxiety (Dibbets and Evers, 2017). Furthermore, the ability to discriminate conditioned stimuli is disturbed in individuals with anxiety; consequently, the ability to recognize cue stimuli associated with a safe stimulus, which is important for the development of the placebo effect, is disturbed in these individuals (Laing et al., 2019). These results suggest that young female participants might have been more anxious than elderly female participants in the present experimental condition, which may impair prediction of the safe stimulus and consequently lead to reduced pain reduction indices (placebo effects) in young female participants.

In concordance, anxiety disturbs prefrontal cognitive function (Park and Moghaddam, 2017). Performance on a working memory task and dl-PFC activity during the task are decreased in patients with social anxiety disorder compared to healthy controls (Balderston et al., 2017a). In healthy participants, anxiety is negatively correlated with working memory capacity (Lukasik et al., 2019). Furthermore, dl-PFC activity is negatively correlated with subjective anxiety (Balderston et al., 2017b), whereas dl-PFC activity decreases with increased activity in the amygdala, which is a critical brain region in anxiety and depression, in participants with high anxiety (Bishop et al., 2007). In addition, increases in amygdala activity have been linked to decreases in dl-PFC activity in healthy participants (Dolcos and McCarthy, 2006). These findings suggest that anxiety may affect dl-PFC activity through emotion-related brain regions, such as the amygdala. These findings suggest that the dl-PFC was less active in the young female group due to anxiety during the task, which might have reduced the pain reduction indices (placebo effects).

Second, in young adults ranging from 18 to 29 years, critical development in the brain may occur particularly in the PFC regions responsible for higher cognitive functions including interference inhibition, decision-making, and response inhibition (Bunge et al., 2002; Scherf et al., 2006; Velanova et al., 2008; Brown et al., 2021). These PFC changes are associated with psycho-social changes of young individuals during this stage known as “emerging adulthood,” distinct from other life stages from a perspective of developmental psychology (Arnett et al., 2014). These findings suggest that these critical PFC changes specific to young females in this stage may alter sensitivity of the dl-PFC to cues associated with safe stimuli. The present results provide neuroscientific evidence that this stage of young adulthood is distinct from other stages especially in females. Further studies are required to investigate the factors such as anxiety or critical PFC changes to affect placebo effects.

In contrast, the effect sizes of the cerebral HbO responses to the sounds in the right dl-PFC were significantly increased in the elderly female group compared to the young female group. Anxiety in female participants decreases with age, and activity in the dl-PFC during working memory tasks is increased in postmenopausal female participants (Jacobs et al., 2017). These results suggest that increased pain reduction indices (placebo effects) due to increased activity in the dl-PFC in the elderly female group may be attributed to decreases in anxiety with age in female participants.

### Limitations

The study is subject to two limitations. First, the small sample size of each group (*n* = 15) may limit the stability of the results. For example, the elderly female group showed smaller VAS pain scores in the LF-LS condition than the elderly male group did, which could be an accidental result of the small sample size. These smaller VAS scores in the LF-LS condition might lead to larger pain reduction indices (placebo effects) in the elderly female group. However, this is unlikely since there were no significant relationships between the VAS pain scores in the LF-LS condition and pain reduction indices (Pearson’s correlation: *r* = –0.209, *p* = 0.109). Second, anxiety levels were not assessed in this study, although anxiety levels could affect placebo effects (see above section “Discussion”). Further studies with larger sample sizes and assessment of participants’ anxiety levels are required to determine whether VAS pain scores in the LF-LS condition are smaller in the elderly female group and whether anxiety levels are elevated in the young female group.

## Conclusion

Previous studies have reported that discrimination of different conditioned stimuli (cue stimuli) associated with low-/high-intensity (non-nociceptive/nociceptive) stimuli is essential for predicting pain intensity and the resulting placebo/nocebo effects, and that such discrimination ability is influenced by sex and age (Labar et al., 2004; Lonsdorf et al., 2015). To investigate the effects of sex and age on placebo and nocebo effects, we analyzed cerebral hemodynamic responses in the dl-PFC using NIRS in the differential conditioning task, in which LF and HF sounds were paired with low- and high-intensity electrical shock, respectively. The results indicated that pain reduction indices (placebo effects) and cerebral HbO responses to the sounds associated with the low- and high-intensity shocks in the right dl-PFC were significantly lower in the young female group than in the elderly female group, whereas there was no age difference in the male groups. Furthermore, pain reduction indices (placebo effects) were significantly correlated with effect sizes of cerebral HbO responses in the right dl-PFC to the LF sound associated with the safe stimulus in all groups except the young female group. These findings suggest that age- and sex-related factors, such as anxiety, may alter the responsiveness of the right dl-PFC to associative cues that predict a safe stimulus.

## Data availability statement

The original contributions presented in this study are included in the article/Supplementary material, further inquiries can be directed to the corresponding author.

## Ethics statement

The studies involving human participants were reviewed and approved by the Ethics Assessment Committee for Research with Humans at the University of Toyama. The patients/participants provided their written informed consent to participate in this study.

## Author contributions

HisN and KT conceived the study and designed the experiment. YI, DH, and KT performed the experiment. YI, DH, KT, and HisN analyzed data and wrote the manuscript. HisN, HirN, JM, and TS revised the manuscript. All authors discussed the results and commented on the manuscript, read, and approved the final manuscript.

## Footnotes

1
http://www.MRIcro.com
