# Supplementary material for: Young female participants show blunted placebo effects associated with blunted responses to a cue predicting a safe stimulus in the right dorsolateral prefrontal cortex
Source: Front Neurosci. 2022 Oct 3;16:1001177. doi: 10.3389/fnins.2022.1001177 (PMC9574021; doi:10.3389/fnins.2022.1001177)
Supplement: Supplementary file 1 [file Data_Sheet_1.PDF]

Young female participants show blunted placebo effects associated with blunted responses to a cue predicting a safe stimulus in the right dorsolateral prefrontal cortex

Iwama et al.

Supplementary information

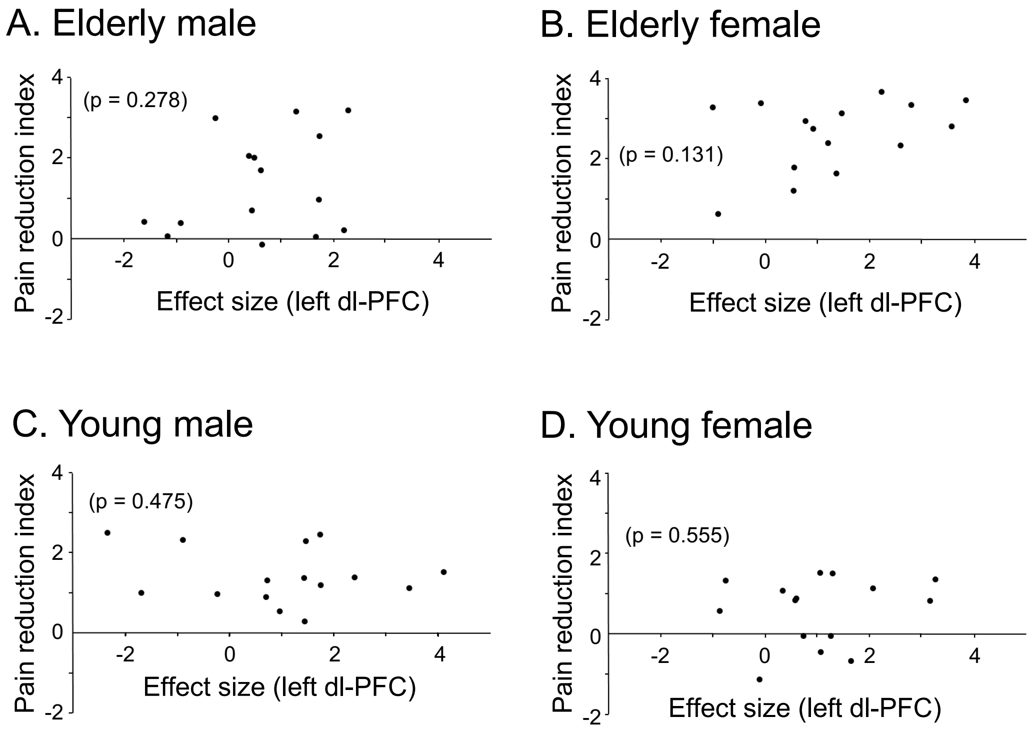

Supplementary Figure 1. Relationships between pain reduction indices and effect sizes of cerebral HbO responses to the LF sound in the left dl-PFC in elderly male (A), elderly female (B), young male (C), and young female (D) groups. No significant correlations are observed in all groups.

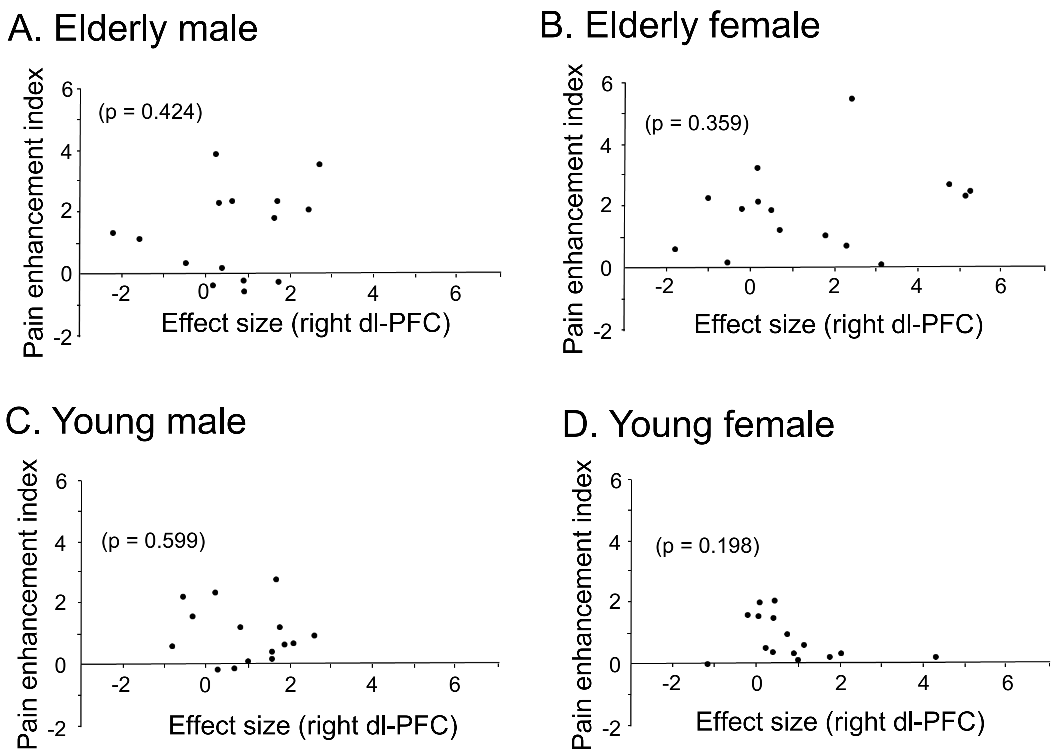

Supplementary Figure 2. Relationships between pain enhancement indices and effect sizes of cerebral HbO responses to the HF sound in the right dl-PFC in elderly male (A), elderly female (B), young male (C), and young female (D) groups. No significant correlations are observed in all groups.

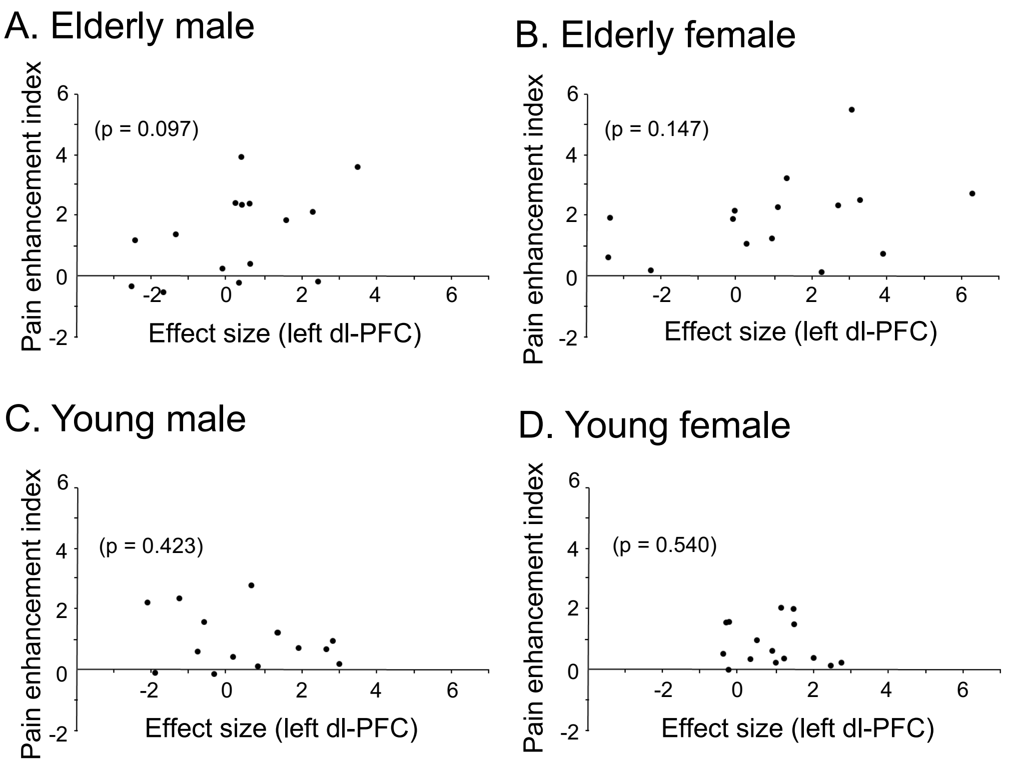

Supplementary Figure 3. Relationships between pain enhancement indices and effect sizes of cerebral HbO responses to the HF sound in the left dl-PFC in elderly male (A), elderly female (B), young male (C), and young female (D) groups. No significant correlations are observed in all groups.
